# Reactivation of mutant p53 by capsaicin, the major constituent of peppers

**DOI:** 10.1186/s13046-016-0417-9

**Published:** 2016-09-06

**Authors:** Alessia Garufi, Giuseppa Pistritto, Mara Cirone, Gabriella D’Orazi

**Affiliations:** 1 Regina Elena National Cancer Institute, Department of Research, Advanced Diagnostics, and Technological Innovation, Unit of Cellular Networks and Molecular Therapeutic Targets, Rome, 00144 Italy; 2 Department of Medical Sciences, Tumor Biology Unit, University “G. d’Annunzio”, Chieti, 66013 Italy; 3 Department of Systems Medicine, University “Tor Vergata”, Rome, 00133 Italy; 4 Department of Experimental Medicine, Istituto Pasteur Fondazione Cenci Bolognetti, University “Sapienza”, Rome, 00161 Italy

**Keywords:** p53, Capsaicin, Autophagy, Apoptosis, Natural compounds, p53 reactivation, Mutant p53

## Abstract

**Background:**

Mutations in the p53 oncosuppressor gene are highly frequent in human cancers. These alterations are mainly point mutations in the DNA binding domain of p53 and disable p53 from transactivating target genes devoted to anticancer activity. Mutant p53 proteins are usually more stable than wild-type p53 and may not only impair wild-type p53 activity but also acquire pro-oncogenic functions. Therefore, targeting mutant p53 to clear the hyperstable proteins or change p53 conformation to reactivate wild-type p53 protein functions is a powerful anticancer strategy. Several small molecules have been tested for p53 reactivation in mutant p53-carrying cells while studies exploiting the effect of natural compounds are limited. Capsaicin (CPS) is the major constituent of peppers and show antitumor activity by targeting several molecular pathway, however, its effect on mutant p53 reactivation has not been assessed yet. In this study we aimed at investigating whether mutant p53 could be a new target of capsaicin-induced cell death and the underlying mechanisms.

**Methods:**

p53 levels were analysed by western blot upon capsaicin treatment in the presence of the autophagy inhibitor chloroquine. The mutant p53 reactivation was evaluated by chromatin-immunoprecipitation (ChIP) assay and semi-quantitative RT-PCR analyses of wild-type p53 target genes. The specific wild-type p53 activation was determined by using the inhibitor of p53 transactivation function, pifithrin-α and siRNA for p53.

**Results:**

Here, we show that capsaicin induced autophagy that was, at least in part, responsible of mutant p53 protein degradation. Abrogation of mutant p53 by capsaicin restored wild-type p53 activities over mutant p53 functions, contributing to cancer cell death. Similar effects were confirmed in cancer cells bearing tumor-associated p53 mutations and in H1299 (p53 null) with overexpressed p53R175H and p53R273H mutant proteins.

**Conclusion:**

These findings demonstrate for the first time that capsaicin may reduce mutant p53 levels and reactivate wild-type p53 protein in mutant p53-carrying cells and the p53 reactivation contributes to capsaicin-induced cell death.

## Background

*TP53* is the major tumor-suppressor gene that encodes for a DNA-binding transcription factor that, upon activation, regulates sequence-specific target genes involved in cell growth inhibition, senescence and apoptosis, providing powerful intrinsic defence against cancer [1]. Thus, an intact p53 pathway protects cells from tumorigenesis, reduces tumor progression, and activates tumor cell response to anticancer drugs [2]. Approximately 55 % of human tumors have loss of wild-type (wt) p53 function mainly due to point mutations in the DNA-binding domain (DBD) ([3, 4], http://p53.iarc.fr), which partially or completely distort p53 protein conformation [5]. These findings indicate that the presence of a functional wtp53 is incompatible with neoplastic cell growth [6]. The major consequence of mutations in the DBD is loss of p53 binding to the canonical sequence-specific target genes with impairment of wtp53 oncosuppressor functions. Mutant p53 (mutp53) often accumulates to high levels in tumors [7] and such hyperstable mutp53 proteins may acquire pro-oncogenic functions contributing to tumor progression and resistance to therapies [8, 9]. Thus targeting mutp53 is a promising strategy for anticancer treatments. Some molecules have been so far shown to target mutp53 for protein degradation or conformation change, providing new insight on mutp53 reactivation [10, 11]. Therefore, the search of novel mutp53-targeting molecules is an emergent field of research due to the important implications in cancer therapy.

Numerous phytochemicals from nature have been investigated for their anticancer activities. Such natural compounds may target multiple signaling pathways and cancer-associated genes; for that reason, several preclinical studies have suggested that natural compounds can also increase the sensitivity of chemoresistant cancers to chemotherapies [12]. In addition, natural compounds are generally less toxic than synthetic drugs. Therefore, a better understanding of their activities and molecular targets is crucial to translate the use of natural compounds in clinic. Capsaicin (8-methyl-*N*-vanillyl-6-noneamide) (CPS) is the major constituent of peppers belonging to the genus *Capsicum* and responsible for their spicy flavor and burning sensation, also known as pungency [13]. Capsaicin has been shown to have antitumor activity in vitro and in vivo; it is able to induce apoptosis through intracellular calcium increase, reactive oxygen species generation, and disruption of mitochondrial membrane transition potential [14]. Moreover, a role of autophagy in capsaicin-triggered cell death has been proposed [15]. Autophagy is a proteolytic process that is activated during various conditions of cellular stress, including nutrient deprivation or DNA damage to eliminate unfolded proteins or damaged organelles to survive bioenergetic stress and/or induce cell death [16]. We have previously shown that autophagy is involved in mutp53 degradation, with the consequence of changing the balance between folded–misfolded p53 proteins and therefore restore wild-type over mutant p53 functions [17, 18]. In this study we aimed at investigating whether mutp53 could be a new target of capsaicin-induced cell death and the underlying mechanisms. We found that CPS-induced cell death in mutant p53-carrying cells was, at least in part, dependent on p53 reactivation, as evidenced by experiments using siRNA interference for p53. Mechanistically, we found that CPS triggered mutp53 degradation likely through autophagy. Moreover, CPS restored wtp53 functions such as DNA binding and transactivation of target genes, that were inhibited by blocking autophagy with chloroquine (CHQ). Inhibiting p53 transactivation with PFT-α impaired CPS-induced wtp53 activation. Finally, CPS improved the cancer cell response to chemotherapeutic drug. These findings demonstrate that mutp53 is a new target of capsaicin-induced cell death.

## Methods

### Cell culture and treatments

In this study human lung cancer cell line H1299 (p53 null) and the glioblastoma cell line U373 (expressing p53 mutation R273H) were maintained in RPMI-1640 (Life Technology-Invitrogen, Carlsbad, CA, USA), while human breast cancer cell line SKBR3 (expressing p53 mutation R175H) was maintained in DMEM (Life Technology-Invitrogen); all cell lines were supplemented with 10 % heat-inactivated fetal bovine serum (FBS), 100 units/mL penicillin, 100 μg/mL streptomycin (Life Technology-Invitrogen), and 2 mmol/LL-glutamine (Life Technology-Invitrogen) in a humidified atmosphere with 5 % CO_2_ and 95 % air at 37 °C.

Unless otherwise indicated, chemicals were from Sigma-Aldrich (St. Louis, MO, USA). Capsaicin (CPS) was dissolved in DMSO and used at 100 and 200 μM; the inhibitor of autophagic protein degradation chloroquine (CHQ) was dissolved in dH_2_O and used at 25 μM; the p53 inhibitor pifithrin-α (PFT) [19] (ENZO Life Sciences, Lausen, Switzerland) was dissolved in DMSO and used at 30 μM; the chemotherapeutic drugs adryamycin (ADR) and cisplatin (CDDP) (Teva, Pharma Italia, Italy) were added to the culture media, respectively, at 1.5 μg/ml and 2.5 μg/ml for the indicated times, as previously reported [20].

### Transfection and vectors

H1299 cells were plated at subconfluence in 30 mm Petri dishes and, the day after, transfected with the expression vectors pcDNA3, pcDNA3-p53R175H and pcDNA3-p53R273H by using the cationic polymer LipofectaminePlus method (Life Technology-Invitrogen), according to the manufacturer’s instructions. The day after transfection cells were treated with CPS for the indicated experiments.

### siRNA interference

U373 and SKBR3 cells were plated at semiconfluence in 35 mm dishes the day before transfection. Control pSuper and pSuper-p53 (for p53 interference, si-p53) vectors [21] were transfected overnight using LipofectaminePlus reagent (Invitrogen) and 24 h later cells were trypsinized and replated for the indicated experiments.

### Cell viability

For viability assay, subconfluent cells were plated in triplicate in 60 mm Petri dishes and 24 h later treated with the indicated reagents, according to dose and time. Both floating and adherent cells were collected and cell viability was determined by Trypan blue exclusion by direct counting with a haemocytometer. The percentage of cell death, as blue/total cells, was assayed by scoring about 200 cells per well in triplicate.

### Chromatin-immunoprecipitation (ChIP) assay

ChIP assay was carried out essentially as previously described [22]. Briefly, protein complexes were cross-linked to DNA in living cells by adding formaldehyde directly to the cell culture medium at 1 % final concentration. Chromatin extracts containing DNA fragments with an average size of 500 bp were incubated overnight at 4^0^ C with milk shaking using polyclonal anti-p53 antibody (FL393, Santa Cruz Biotechnology). Before use, protein G (Pierce) was blocked with 1 μg/μL sheared herring sperm DNA and 1 μg/μL BSA for 3 h at 4^0^ C and then incubated with chromatin and antibodies for 2 h at 4^0^ C. PCR was performed with HOT-MASTER Taq (Eppendorf) using 2 μL of immuniprecipitated DNA and promoter-specific primers. Immunoprecipitation with non-specific immunoglobulins (IgG; Santa Cruz Biotechnology) was performed as negative controls. The amount of precipitated chromatin measured in each PCR was normalized with the amount of chromatin present in the input of each immunoprecipitation. PCR products were run on a 2 % agarose gel and visualized by ethidium bromide staining using UV light.

### RNA extraction and semi-quantitative reverse transcription (RT)-PCR analysis

Cells were harvested in TRIzol Reagent (Invitrogen) and total RNA was isolated following the manufacturer’s instructions. The first strand cDNA was synthesized from 2 μg of total RNA with MuLV reverse transcriptase kit (Applied Biosystems). Semi-quantitative Reverse-Transcribed (RT)-PCR was carried out by using Hot-Master Taq polymerase (Eppendorf) with 2 μl cDNA reaction and genes specific oligonucleotides under conditions of linear amplification. PCR products were run on a 2 % agarose gel and visualized with ethidium bromide. The housekeeping 28S gene, used as internal standard, was amplified from the same cDNA reaction mixture. Densitometric analysis was applied to quantify mRNA levels compared to control gene expression.

### Western blotting

Total cell extracts were prepared by incubation in lysis buffer (50 mM Tris–HCl, pH 7.5, 150 mM NaCl, 5 mM EDTA, 150 mM KCl, 1 mM dithiothreitol, 1 % Nonidet P-40) and a mix of protease inhibitors and resolved by 9–18 % SDS-polyacrilamide gel electrophoresis. Proteins were transferred to a polyvinylidene difluoride membrane (PVDF, Millipore) and membranes were blocked with 5 % nonfat dry milk in PBS and incubated with the following primary antibodies: monoclonal anti-poly (ADP-ribose) polymerase (PARP, BD Pharmingen, CA, USA), monoclonal anti-p53 (Ab-DO1), polyclonal anti-p53 (FL393), monoclonal anti-p62 (Santa Cruz Biotechnology, Dallas, TX, USA), monoclonal anti-phospho-Histone H2AX (Ser139) (Millipore, clone JBW301), and monoclonal anti-LC3B (Sigma-Aldrich). Equal lane loading was monitored by probing membranes with antibodies specific for β-actin (Calbiochem, San Diego, CA, USA). Primary antibodies were detected with appropriate horseradish peroxidase-labeled secondary antibodies (Bio-Rad, Hercules, CA, USA). Enzymatic signals were visualized using chemoluminescence (ECL Detection system, GE Healthcare, Milan, Italy).

### Statistical analyses

Each experiment, unless specified, was performed at least three times. Results are expressed as values of mean ± standard deviation (S.D.). Statistical significance was determined using Student’s *t*-tests for two sample comparisons and one-way ANOVA analysis for three or more sample comparisons.

## Results

### Effect of capsaicin on proliferation of cells expressing mutant p53

**Fig. 1:**
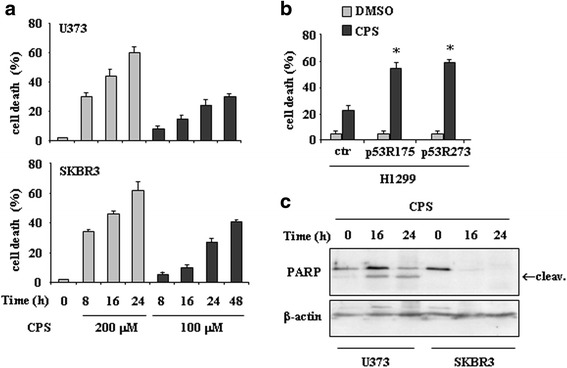
CPS induces cell death in mutp53-expressing cells in a p53-dependent manner. **a** U373 and SKBR3 cells were plated at subconfluence and the day after treated with CPS (100 and 200 μM). Twenty-four hours later, the percentage of dead cells was scored by trypan blue staining. Error bars show standard deviation. **b** H1299 cells were transiently transfected with pcDNA3-p53R175H (0.1 μg), pcDNA3-R273H (0.1 μg) and control pcDNA3 vectors and the day after transfection treated with CPS (200 μM) for 24 h. Cell death measurements were assayed by Tunel assay. The results are the mean of three independent experiments performed in triplicate ± SD. *p = 0.001. **c** U373 and SKBR3 cells were treated with CPS (200 μM) for 16 and 24 h. Equal amount of total cell extracts was analysed by western immunoblotting with anti-PARP antibody; the cleaved form is indicated by the arrow. Anti-β-actin was used as protein loading control

We first examined the biological effect of capsaicin (CPS) in tumor cells harbouring mutations at the hotspot codons 175 (that is, SKBR3) and 273 (that is, U373). We performed a dose–response analysis to analyse cell viability by trypan blue exclusion. As shown in Fig. 1a, both doses of CPS used (100 and 200 μM) induced cell death in time-dependent manner, although the extent of cell death was higher at 200 μM dose. Similarly, H1299 cells transfected with p53-R175 and p53-R273 plasmids displayed increased sensitivity to CPS, compared to H1299 cells transfected with control vector (ctr) (Fig. 1b). Western immunoblotting revealed PARP cleavage as soon as 16 h after treatment in both cell lines, indicating the occurrence of apoptotic cell death (Fig. 1c).

**Fig. 2:**
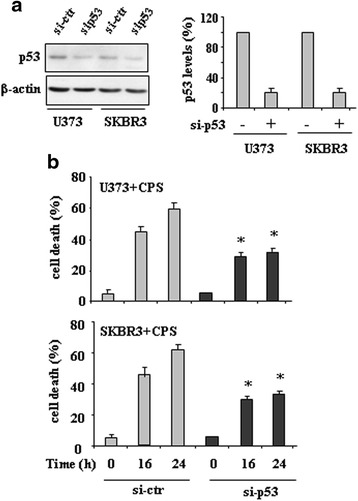
p53 interference reduces the CPS-induced cell death in mutp53-expressing cancer cells. **a** U373 and SKBR3 were transfected with pSuper (si-ctr) or pSuper-p53 (si-p53) and 36 h after transfection equal amount of total cell extracts was analysed by western immunoblotting with anti-p53 antibody. Anti-β-actin was used as protein loading control. The percentage of p53 reduction was measured by densitometry and plotted in the right panel. **b** Control and interfered cells as in (**a**) were treated with CPS (200 μM). Twenty-four hours later, the percentage of dead cells was scored by trypan blue staining. Data are the mean ± S.D. of three independent experiments performed in triplicate. *P = 0.001

To determine whether the antiproliferative effect of CPS was mediated by reactivation of mutp53, SKBR3 and U373 cells were transfected with control si-RNA (si-ctr) or p53 siRNA (si-p53) expressing vectors. The p53 protein was reduced by ≥80 % after p53 siRNA transfection (Fig. 2a). As shown in Fig. 2b, mutp53 knockdown strongly reduced the sensitivity to CPS-induced cell death, whereas si-ctr cells remained highly sensitive. These results demonstrate that CPS-induced cell death in mutp53-carrying cells was in part dependent on p53 reactivation.

### CPS triggers mutant p53 protein degradation

**Fig. 3:**
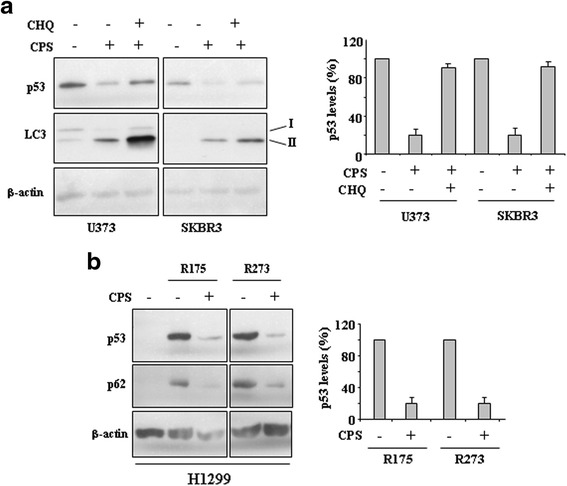
CPS induces mutp53 protein degradation. **a** U373 and SKBR3 cells were treated with CPS (200 μM) and chloroquine (CHQ) (25 μM) for 24 h. Protein levels were measured with western blot using antibodies to LC3-II and p53. Anti-β-actin was used as protein loading control. In the right panel data are presented as mean ± S.D. *P = 0.001. **b** H1299 cells were transiently transfected with pcDNA3-p53R175H (0.1 μg), pcDNA3-R273H (0.1 μg) and control pcDNA3 vectors and the day after transfection treated with CPS (200 μM) for 24 h. Cell death measurements were assayed by Tunel assay. Equal amount of total cell extracts was analysed by western immunoblotting with anti-p53 and anti-p62 antibodies. Anti-β-actin was used as protein loading control. The percentage of p53 reduction was measured by densitometry and plotted in the right panel. *P = 0.001

Small-molecule compounds targeting mutp53 have been recently used on the basis of putative conformational changes within mutp53 proteins to restore wild-type p53, or protein degradation through autophagy, as also demonstrated by our studies [17, 18]. Therefore, we investigated whether CPS could trigger mutp53 protein clearing. To this aim we evaluated the expression of microtubule-associated protein light chain 3 (LC3) protein that, after conversion from LC3-I to its autophagosome membrane-associated lipidated form LC3-II, is considered a cellular readout of autophagy [23]. As shown in Fig. 3a, CPS increased LC3-II protein levels and, parallel to induction of autophagy, triggered mutp53 downregulation in both cell lines, as assessed by densitometric analysis (Fig. 3a, right panel). The use of chemical inhibitor of autophagic/lysosomal degradation chloroquine (CHQ) [23] prevented CPS-induced mutp53 degradation (Fig. 3a and b), underlining the role of autophagy in mutp53 degradation. In agreement, CPS induced mutp53 degradation in H1299 cells transfected with p53-R175 and p53-R273 plasmids, along with reduction of p62 levels as readout of autophagy induction [23] (Fig. 3b). Altogether, these findings indicate that CPS induced mutp53 degradation through autophagy.

### CPS restores wtp53 functions such as DNA binding and transactivation of target genes

**Fig. 4:**
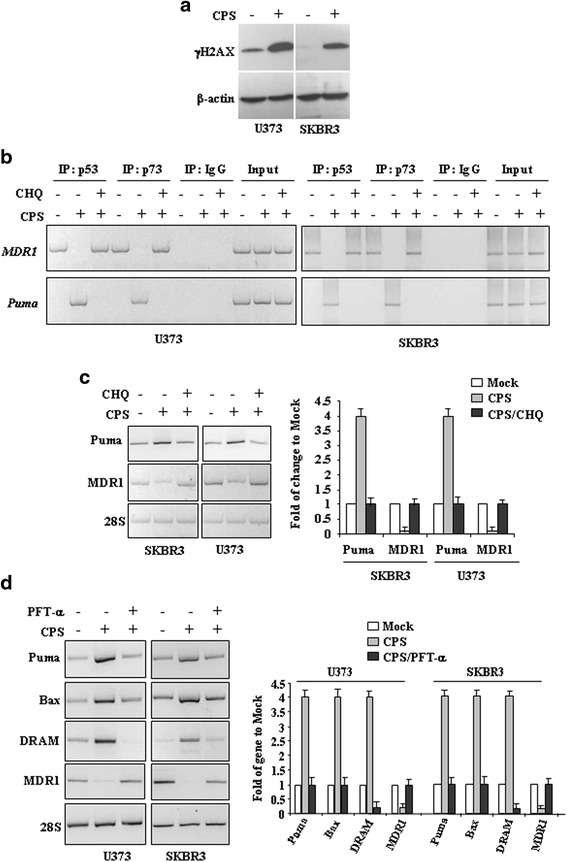
CPS restores wtp53 activities in mutp53-carrying cells. **a** U373 and SKBR3 were treated with CPS (200 μM) for 24 h. Western immunoblotting was performed on equal amount of total cell extracts to detect phospho-Histone H2A.X (γH2AX) levels. Anti-β-actin was used as protein loading control. **b** SKBR3 and U373 cells (6x10^6^) were plated in 150 mm dish and the day after treated with CPS (200 μM) for 16 h before being assayed for chromatin immunoprecipitation analysis (ChIP) with anti-p53 or anti-p73 antibodies. PCR analyses were performed on the immunoprecipitated DNA samples using primers specific for wtp53 target gene promoter (Puma) or for mtp53 target promoter (MDR1). A sample representing linear amplification of the total chromatin (Input) was included as control. Additional controls included immunoprecipitation performed with non-specific immunogloblulins (No Ab). SKBR3 and U373 cells were plated at subconfluence in 60 mm dish and the day after treated with CPS (200 μM) for 24 h, with or without autophagy inhibitor chloroquine (CHQ) (**c**) or p53 inhibitor pifithrin-α (PFT-α) (30 μM) (**d**). p53 target genes were detected by RT-PCR analysis. β-actin was used as control. Gene expression was measured by densitometry, normalized to β-actin levels, ±SD (right panels) and plotted as fold of mRNA expression over control (Mock)

To determine whether CPS has chemical genotoxicity that may be involved in wtp53 activation, we examined the phosphorylation of the subtype of histone H2A, called H2AX, in the position of Ser139 producing γH2AX, a marker of DNA double-strand breaks [24]. Herein we found that CPS treatment produced γH2AX expression in both cell lines (Fig. 4a), therefore, we determined reactivation of wtp53 functions upon CPS, by chromatin immunoprecipitation (ChIP) assay and analysis of target gene transcription. Binding to sequence specific DNA promoters is critical for wtp53 oncosuppressor functions [25]. The results show that CPS restored p53-DNA binding activity to the wild-type target gene promoter *PUMA* to the detriment of mutp53-activated promoter *MDR1* [26] (Fig. 4b). In agreement with the CPS-induced mutp53 autophagic degradation, the use of CHQ reversed such DNA binding. We also performed ChIP analyses using the p73 antibody because one of the mutp53 oncogenic characteristics is to bind the family member p73 with inactivation of p73 pro-apoptotic function [27]. Parallel to p53 results, ChIP analyses revealed that the p73 recruitment onto target promoters was induced after CPS treatment and reversed by CHQ (Fig. 4b), mirroring that of reactivated mut/wtp53. The role of autophagy in CPS-induced p53 reactivation was evaluated by measuring the transcription of endogenous p53 target genes. The results show that the CPS-induced wtp53 target gene *Puma* was efficiently reverted by concomitant inhibition of autophagy with chloroquine (CHQ) (Fig. 4c); in agreement, the reduction of the mutp53 target gene *MDR1* by CPS was reverted by CHQ (Fig. 4c), in agreement with the mutant/wild-type balance change. In addition, the specific effect of CPS in reactivating p53 transactivation function was finally evaluated by using the inhibitor of wtp53 transactivation function PFT-α [19]. The results show that PFT-α indeed impaired the increase of wtp53 target genes *PUMA*, *Bax*, and *DRAM* in SKBR3 and U373 cells after CPS treatment and restored the transcription of the mutp53 target gene *MDR1* (Fig. 4c). Altogether, these results demonstrate that CPS restores wtp53 transcriptional activity in mutp53-carrying cancer cells.

### CPS improves the drugs-induced tumor cell death

**Fig. 5:**
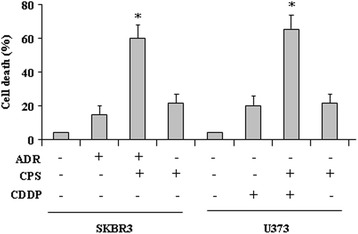
CPS increases drug-induced cell death in mutp53-carrying cells. SKBR3 and U373 cells were plated at subconfluence and the day after treated with CPS (100 μM) and, respectively, with ADR (1.5 μg/ml) and cisplatin (CDDP, 2.5 μg/ml). Twenty-four hours later, the percentage of dead cells was scored by trypan blue staining. Error bars show standard deviation. *P = 0.001

Finally, we evaluated the biological outcome of CPS in combination with chemotherapeutic agents. To this aim, SKBR3 and U373 cells were treated, respectively, with adryamycin (ADR, 1.5 μg) and cisplatin (CDDP, 2.5 μg) alone or in combination with CPS (100 μM) for 24 h, as previously reported [20]. The results show that the slight cell death effect of ADR or CDDP and CPS alone was significantly increased when the chemotherapeutic drugs were used in combination with CPS (Fig. 5), suggesting that CPS-induced p53 reactivation may improve mutp53-carrying cancer cell response to chemotherapy.

## Discussion

Mutp53 proteins may drive tumor progression, metastasis and resistance to therapies [4], leading to poorer patient outcomes [28]. Therefore, reactivation of mutp53 proteins holds great promises in cancer therapy [29]. As a proof of principle, clearing of mutp53 has been shown to reduce tumor malignancy and to impair mutp53 gain-of-function (GOF), thus improving the apoptotic response to drugs [30, 31]. In addition, as mutp53 proteins exert a dominant negative effect on wtp53 [32], changing the balance between folded–misfolded p53 proteins may restore wild-type over mutant p53 functions, as also reported by our previous studies [17, 18, 33]. Restoration of wtp53 activity is extremely helpful for eradicating established tumors [34–36] and does not damage nontransformed cells [37]. Several small molecules have been tested for p53 reactivation in mutp53-carrying cells [10, 11, 38] while studies exploiting the effect of natural compounds are limited. Here, we show that CPS, the major constituent of peppers, induced mutp53 protein degradation, in part through autophagy, and that such abrogation restored wild-type p53 activities over mutant p53 functions.

Capsaicin has been used medicinally for centuries, but recently it has been extensively studied for its analgesic, antioxidant, anti-inflammatory, and anti-obesity properties; moreover, anti-cancer and pro-apoptotic effects have also been reported [1, 13, 14, 39–42]. Recently, CPS has also been shown to induce autophagy that eventually ends in cell death [15]. In line with the biological effects of CPS, we found that mutp53 underwent autophagy-mediated protein degradation upon CPS treatment, as confirmed by the use of autophagy inhibitor chloroquine that indeed counteracted the CPS degradative effect on mutp53 restoring the DNA binding and transactivation activities proper of wtp53 protein. In response to CPS indeed, p53 transactivated apoptotic genes such as *Puma*, *Bax* and *DRAM* to the detriment of mutp53-activated promoter *MDR1*. In addition, *DRAM* (damage-regulated autophagy modulator) has been shown to induce autophagy that, in this setting, may sustain mutp53 degradation, as we previously reported [17], thus contributing to p53-dependent apoptosis [43]. The consequence was p53-dependent cancer cell death in response to CPS. In addition, CPS improved the cell death response in combination with chemotherapeutic drug. Such effect could be exploited to reduce the amount of chemotherapy with the aim to reduce the toxic side effects, as we previously showed for the natural compound zinc in combination with drugs [44]. Few natural compounds have been so far found to induce mutp53 degradation [17, 18, 45, 46], and, to the best of our knowledge, this is the first time that CPS is shown to degrade mutp53 protein and reactivate wtp53 in mutp53-carrying cell lines.

Successful anticancer therapy is achieved when cancer cells undergo apoptosis and p53 is the major player in apoptosis induction against cancer cells [47], therefore, restoring wtp53 activity plays a fundamental role in anticancer strategies. The role of CPS in reactivating wild-type p53 functions in mutp53-carrying cells was demonstrated here by the use of the inhibitor of wtp53 transactivation function, pifithrin-α (PFT-α) [19] that indeed impaired the induction of wtp53 target genes after CPS treatment. Once the balance between folded-misfolded p53 protein is changed to increase the wtp53 proteins, p53 can be activated. p53 is activated in response to a variety of stress signals, including DNA damage, hypoxia and aberrant proliferation signals such as oncogene activation [2]. Here, CPS was shown to induce γH2AX that in general occurs in response to formation of double strand brakes (DSB) and is an early sign of replication stalling [24]. In agreement, CPS has been shown to activate wtp53 that therefore contributes to CPS-induced cell death [48, 49]. For the past several decades, phytochemicals found in fruits, vegetables, whole grain, spices and teas have been hot topics in the area of chemoprevention because they exhibit a number of inhibitory effects against cancer initiation, promotion, progression and metastasis [50]. Moreover, phytochemicals are readily available, inexpensive and generally non-toxic, which gives them an advantage with respect to pharmaceutical drugs which are indiscriminately toxic for patient undergoing therapy and show side-effects and drug resistance. In conclusion, we show here for the first time that CPS is able to target mutp53 proteins and in particular R175 mutation that is the third most common missense p53 mutation in human cancer and R273 that is one of the p53 mutation responsible of resistance to antitumor drugs [4], reactivating wild-type p53 oncosuppressor function. These findings could be the used to exploit the activity of CPS to develop novel prodrugs that could specifically target mutp53-carrying tumors.

## Abbreviations

ADR: Adryamycin
CDDP: Cisplatin
ChIP: Chromatin-immunoprecipitation
CHQ: Chloroquine
CPS: Capsaicin
DBD: DNA-binding domain
FBS: Fetal bovine serum
IgG: Immunoglobulins
LC3: Light chain 3
mutp53: Mutant p53
PARP: Poly (ADP-ribose) polymerase
PFT-α: Pifithrin-α
RT-PCR: Reverse transcription polymerase chain reaction
SD: Standard deviation
siRNA: Small interference RNA
wtp53: Wild-type p53
